# From Sample to Multi-Omics Conclusions in under 48 Hours

**DOI:** 10.1128/mSystems.00038-16

**Published:** 2016-04-26

**Authors:** Robert A. Quinn, Jose A. Navas-Molina, Embriette R. Hyde, Se Jin Song, Yoshiki Vázquez-Baeza, Greg Humphrey, James Gaffney, Jeremiah J. Minich, Alexey V. Melnik, Jakob Herschend, Jeff DeReus, Austin Durant, Rachel J. Dutton, Mahdieh Khosroheidari, Clifford Green, Ricardo da Silva, Pieter C. Dorrestein, Rob Knight

**Affiliations:** a Collaborative Mass Spectrometry Innovation Center, Skaggs School of Pharmacy and Pharmaceutical Sciences, University of California, San Diego, San Diego, California, USA; b Department of Pediatrics, University of California, San Diego, San Diego, California, USA; c Department of Computer Science and Engineering, University of California, San Diego, San Diego, California, USA; d Fermenters Club of San Diego, San Diego, California, USA; e Division of Biological Sciences, University of California, San Diego, San Diego, California, USA; f Institute for Genomic Medicine Genomics Center, University of California, San Diego, San Diego, California, USA; g Center for Microbiome Innovation, University of California, San Diego, San Diego, California, USA; h Department of Pharmacology, University of California, San Diego, San Diego, California, USA; i Department of Ecology and Evolutionary Biology, University of Colorado Boulder, Boulder, Colorado, USA; Argonne National Laboratory

**Keywords:** 16S rRNA, microbiome, fermented food, metabolome, molecular networking, rapid response

## Abstract

Multi-omics methods have greatly advanced our understanding of the biological organism and its microbial associates. However, they are not routinely used in clinical or industrial applications, due to the length of time required to generate and analyze omics data. Here, we applied a novel integrated omics pipeline for the analysis of human and environmental samples in under 48 h. Human subjects that ferment their own foods provided swab samples from skin, feces, oral cavity, fermented foods, and household surfaces to assess the impact of home food fermentation on their microbial and chemical ecology. These samples were analyzed with 16S rRNA gene sequencing, inferred gene function profiles, and liquid chromatography-tandem mass spectrometry (LC-MS/MS) metabolomics through the Qiita, PICRUSt, and GNPS pipelines, respectively. The human sample microbiomes clustered with the corresponding sample types in the American Gut Project (http://www.americangut.org), and the fermented food samples produced a separate cluster. The microbial communities of the household surfaces were primarily sourced from the fermented foods, and their consumption was associated with increased gut microbial diversity. Untargeted metabolomics revealed that human skin and fermented food samples had separate chemical ecologies and that stool was more similar to fermented foods than to other sample types. Metabolites from the fermented foods, including plant products such as procyanidin and pheophytin, were present in the skin and stool samples of the individuals consuming the foods. Some food metabolites were modified during digestion, and others were detected in stool intact. This study represents a first-of-its-kind analysis of multi-omics data that achieved time intervals matching those of classic microbiological culturing.

**IMPORTANCE** Polymicrobial infections are difficult to diagnose due to the challenge in comprehensively cultivating the microbes present. Omics methods, such as 16S rRNA sequencing, metagenomics, and metabolomics, can provide a more complete picture of a microbial community and its metabolite production, without the biases and selectivity of microbial culture. However, these advanced methods have not been applied to clinical or industrial microbiology or other areas where complex microbial dysbioses require immediate intervention. The reason for this is the length of time required to generate and analyze omics data. Here, we describe the development and application of a pipeline for multi-omics data analysis in time frames matching those of the culture-based approaches often used for these applications. This study applied multi-omics methods effectively in clinically relevant time frames and sets a precedent toward their implementation in clinical medicine and industrial microbiology.

## Abstract (precis)

Polymicrobial infections are difficult to diagnose due to the challenge in comprehensively cultivating the microbes present. Omics methods, such as 16S rRNA sequencing, metagenomics, and metabolomics, can provide a more complete picture of a microbial community and its metabolite production, without the biases and selectivity of microbial culture. However, these advanced methods have not been applied to clinical or industrial microbiology or other areas where complex microbial dysbioses require immediate intervention. The reason for this is the length of time required to generate and analyze omics data. Here, we describe the development and application of a pipeline for multi-omics data analysis in time frames matching those of the culture-based approaches often used for these applications. This study applied multi-omics methods effectively in clinically relevant time frames and sets a precedent toward their implementation in clinical medicine and industrial microbiology.

## INTRODUCTION

The omics field is expanding rapidly, driven by the plummeting cost of DNA sequencing, the widespread availability of DNA sequencers and mass spectrometers, and the seemingly unlimited breadth of its applications. However, generating, processing, analyzing, and interpreting the data typically takes months and requires substantial technical expertise in large multidisciplinary teams, in part, due to the rapidly evolving nature of the component techniques. The speed of mass spectrometry and nucleic acid sequencing (the tools required to generate omics data) has increased rapidly in the last decade, and they have separately been applied to clinical diagnostics in a targeted fashion. For example, high-throughput sequencing for the detection and typing of single pathogens in complex samples has achieved turnaround times of hours to days (1–5), and mass spectrometry analysis of metabolites has been performed in the clinic and laboratory in essentially real time (6, 7). However, the integration of multi-omics technologies and their application to the microbiome field have not yet achieved time frames compatible with clinical needs in human health, industrial microbiology, or routine laboratory experiments.

Multi-omics studies of the human microbiome can have enormous impact, providing a more comprehensive picture of a microbial community than a single omics approach on its own (8, 9). These studies have led to an understanding of how microbial communities in our bodies produce metabolites that affect our health and transform the drugs we consume (10–13). One of the first integrated omics analysis related to the human microbiome was by Li et al. (14), who revealed an association between the gut microbiota and host metabolites in a cohort of Chinese subjects by using clone library sequencing and nuclear magnetic resonance. This, and more recent multi-omics studies (15, 16), had multiyear gestation times. Today, when considering the time between receipt of samples with informed consent and statistical conclusions from integrated omics data, these studies still require months to years to complete.

In order to develop rapid multi-omics pipelines with broad applicability, they must first be tested using subjects and samples that are strongly influenced by their exposure to microbes and microbial chemical products. The subjects in this study are tightly linked to their microbial partners through their active involvement with fermented foods. This mutualistic relationship is believed to have existed since the Paleolithic era (17) and continues around the globe today. Modern human evolution is intertwined with the influence of microbial fermentation processes in the foods we eat and within our own bodies. Depending on the type of food and conditions used during fermentation, different types of microbial communities form, composed of various bacterial and fungal species (18), and the metabolic products of these communities can impact human health (19). Previous studies found that species originating from microbially diverse fermented foods, such as cheese and salami, are able to colonize the gastrointestinal tract (19). Furthermore, with the significant effects of antibiotics and a processed food-based diet on our microbiomes (13, 20, 21), there is an interest in the health benefits of fermented foods as alternatives. Here, we present the results from a simple, robust multi-omics platform integrating analyses of human, environmental, and animal samples in the clinically relevant time frame of less than 48 h. This pipeline is now possible because of rapid advances in the development of software for the analysis and integration of omics data and standardized protocols that allow streamlined insertion of matched samples into multi-omics pipelines. We demonstrate how individuals commonly exposed to fermented foods show influences of these microbes on and in their bodies.

## RESULTS AND DISCUSSION

### General description of the 48-h analysis and multi-omics pipeline.

**FIG 1:**
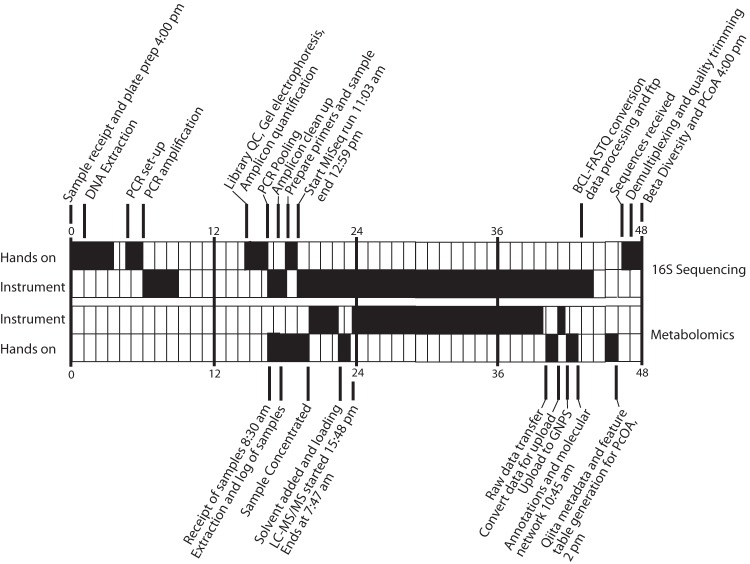
Timeline of the multi-omics analysis of samples from four households and their fermented food products.

Samples were collected by seven volunteers (two families and two individuals, designated households 1 to 4) who regularly prepare and eat fermented foods (specifics of the fermented foods are presented in Table S1 in the supplemental material) and who were recruited to the American Gut Project (AGP; http://www.americangut.org) via word-of-mouth through the Second Annual San Diego Fermentation Festival in San Diego, CA. The AGP is an IRB-approved citizen science project comprising more than 7,000 samples from more than 6,500 individuals. Consenting participants received an AGP sampling kit after they gave consent and took a survey online, and the data were stored in a secure database. The deidentified metadata were then immediately downloaded into a file formatted for use in Qiita (https://qiita.ucsd.edu/). Due to the infrastructure surrounding the process, participant consent and sample-associated metadata were obtained before the samples arrived in the laboratory, facilitating immediate preparation for sample processing upon arrival. Notably, the metadata can be used for both 16S rRNA gene sequencing and metabolomics analyses, further streamlining the multi-omics approach. Samples were collected by cotton swab and subjected to DNA and metabolite extraction to describe the composition and activity of the corresponding microbial communities. Samples were subjected to a streamlined, high-throughput process involving preparation for 16S rRNA gene (variable region 4 [V4]) sequencing via the Earth Microbiome Project protocols (22, 23) and for liquid chromatography-tandem mass spectrometry (LC-MS/MS) (24). The first description of both the microbial communities and molecules, including alpha and beta diversity, and specific effects of fermented foods on the microbial and chemical ecology of the subjects, occurred within 48 h after samples were delivered to the laboratory (Fig. 1). Computational resources, including the Barnacle cluster available through the UCSD center for microbiome innovation connected to the Comet supercomputer located at the San Diego Supercomputer Center, allowed >50 central processing unit (CPU) h of processing in <11 h of wall time (note that some of the component steps are not parallelized), giving results back to the researchers fast enough to interpret the data in a timely manner.

10.1128/mSystems.00038-16.2 Table S1  Specific fermented foods produced and consumed in the households. Download Table S1, DOCX file, 0.04 MB. Copyright © 2016 Quinn et al. 2016 Quinn et al. This content is distributed under the terms of the Creative Commons Attribution 4.0 International license.

There are four main components that enabled the development of this rapid multi-omics pipeline and its implementation in less than 48 h (Fig. 1). First, subjects easily and efficiently enrolled themselves as part of an already existing, IRB-approved project (the AGP), enabling the use of on-the-spot informed consent and standardized metadata collection. Second, the protocols used to collect metadata and process samples have been extensively benchmarked and standardized (http://www.earthmicrobiome.org/emp-standard-protocols/), allowing rapid assimilation with existing datasets and facilitating meaningful comparisons with other cohorts. Third, community analysis infrastructures, including Qiita, the microbial analysis infrastructure that houses microbiome analysis tools, and GNPS, a crowdsourced analysis infrastructure and public metabolomics knowledge repository (http://gnps.ucsd.edu), allowed rapid data processing and interpretation. And fourth, the servers that host Qiita and GNPS are linked, enabling normalization, processing, and cross-platform analysis of multi-omics data in an integrated fashion. Both these analysis platforms enable rapid comparisons to existing data in the public domain and are publicly available, facilitating data upload and analysis from any sequencer or tandem mass spectrometer, so long as the file formats are compatible. Linking the two platforms limits the need to move gigabytes or terabytes of data, making local analysis on one’s own computer and integration with existing knowledge possible, rather than needing to download public data and new data to a personal computer first (e.g., the AGP data repository contains over 216 million reads). Tools available through this pipeline and utilized in this study include operational taxonomic unit (OTU) clustering of reads and generation of tables for multivariate statistical analysis of microbiome data, including alpha diversity, principle component analysis (PCoA) visualization through EMPeror, cluster significance testing with analysis of similarity (ANOSIM), and others. This pipeline also allows immediate integration of data with the data in the AGP repository to visualize the relationships of samples with a large reference data set, which can provide context to the microbiome data generated. Metabolomics tools include library searching of the GNPS libraries (the largest currently available in the mass spectrometry field) (25), molecular network visualization to allow metabolite tracking, and metabolome abundance matrix generation to allow similar multivariate statistical analysis, including PCoA and EMPeror-based visualization of sample relationships.

### Microbiome relationships.

**FIG 2:**
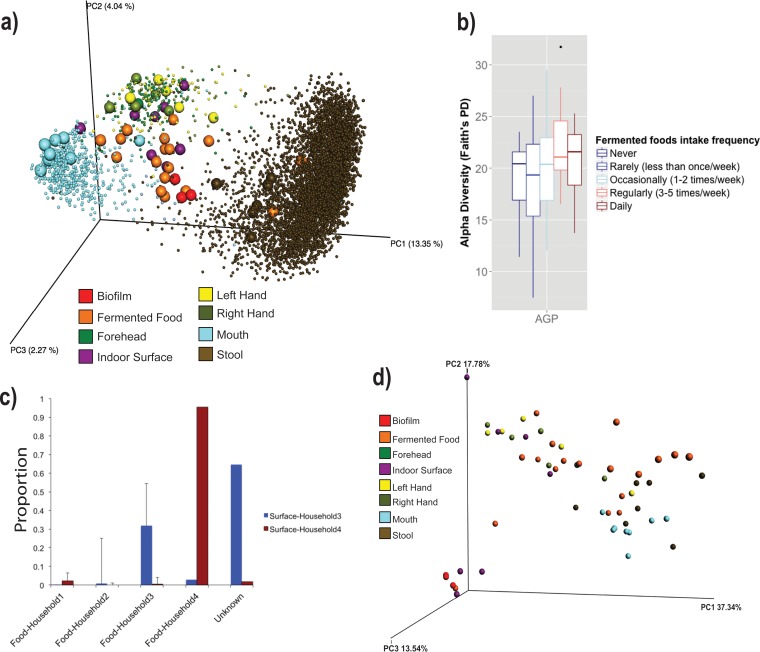
(a) PCoA of the abundance of unique OTUs per sample from the 16S marker gene sequencing data from the AGP data repository (small spheres) and the San Diego Fermentation Festival volunteer samples collected for this study (large spheres). (b) Alpha diversity as measured using 16S rRNA marker gene sequencing counts of OTUs in a subset of the American Gut Project data for which consumption of fermented foods is reported. (c) SourceTracker analysis of surface samples from households 3 and 4. SourceTracker measures the proportions of OTUs sourced from the fermented foods on the household surfaces where they were prepared. (d) PCoA clustering of microbiome data after metagenomic prediction with the PICRUSt algorithm.

Bacterial marker gene sequencing revealed rich microbial communities in most fermented food samples as judged by Faith’s phylogenetic diversity (PD) metric (26), a biodiversity measure incorporating phylogenetic differences between the taxa present in a sample. The three most diverse samples were pickles, beet kvass, and port wine (PD values of 23.0, 16.6, and 16.2, respectively), while dairy kefir and “symbiotic colony of bacteria and yeast” (SCOBY) samples were the least diverse (average PD values of 2.21 and 1.91, respectively). The average PD of all fermented foods in the data set was 9.89, compared to 21.6, 11.9, and 18.5 for human skin, oral, and fecal samples, respectively. Surface microbiomes were also rich, with an average PD of 11.5. The unweighted UniFrac matrix (27) visualized via principle component analysis (PCoA) using EMPeror clustered the samples closely by type (ANOSIM R statistic = 0.477, *P* = 0.001; see Fig. S1 in the supplemental material), and the human sample types matched their corresponding AGP sample types (Fig. 2a). While mouth, stool, and right and left hand samples each formed relatively tight clusters, as expected (28), fermented food and indoor surface samples formed a looser cluster together, largely distinct from human sample clusters, although a few food and surface samples clustered near hand and fecal samples (Fig. 2a). Combining these samples with a subset of the AGP cohort revealed that there was an increase in gut bacterial diversity that correlated with an increase in fermented food consumption (*R*^2^ = 0.034, *P* = 0.02373) (Fig. 2b). Nonparametric Kruskal-Wallis tests corrected for multiple comparisons (false discovery rate [FDR]) identified 219 OTUs differing significantly in relative abundance across sample types. No OTU was significantly higher in fermented food samples than in any other sample type, though several were higher (FDR corrected *P* < 0.05) in stool (including OTUs classified as *Blautia*, *Varibaculum*, *Bacteroides*, *Peptoniphilus*, and *Corynebacterium*), hand (*Corynebacterium*, *Staphylococcus*, *Neisseria*, *Haemophilus*, and *Rothia*), and mouth (*Prevotella*, *Neisseria*, *Lautropia*, and *Leptotrichia*) samples. SourceTracker (29) analysis revealed that the microbial communities of items on or in which fermented foods were prepared (i.e., from surfaces, such as cutting boards, to containers, such as fermenters) were largely sourced from the foods and specific to the location in which the foods were prepared. Except for one household, where small percentages (9 to 30%) of hand microbial communities were sourced from food, no obvious patterns linked microbial source communities to human skin, mouth, or fecal microbiomes (Fig. 2c).

10.1128/mSystems.00038-16.1 Figure S1  PCoA plot of 16S rRNA marker gene sequencing data from food, human, and environmental samples collected in this study. Download Figure S1, PDF file, 0.1 MB. Copyright © 2016 Quinn et al. 2016 Quinn et al. This content is distributed under the terms of the Creative Commons Attribution 4.0 International license.

PICRUSt metagenome predictions revealed a slightly dissimilar clustering pattern to that observed with 16S marker gene sequencing data based on sample type when the Bray-Curtis distance metric was applied to the BIOM table containing KEGG pathways. While fermented food and surface samples still formed a loose cluster, with body types more tightly clustered, oral samples clustered close to fecal samples based on KEGG pathways but not 16S marker gene data (Fig. 2d). Nonparametric Kruskal-Wallis tests corrected for multiple comparisons (FDR) identified 119 KEGG pathways differing significantly across sample types. KEGG pathways that were significantly higher (FDR-corrected *P* value of <0.05) in fermented foods than on surfaces included aminosugar and nucleotide sugar metabolism, starch and sucrose metabolism, galactose metabolism, RNA transport, glycolysis/gluconeogenesis, and methane metabolism; KEGG pathways that were significantly higher on surface samples than in food samples included bacterial secretion systems, phenylalanine metabolism, fluorobenzoate degradation, aminobenzoate degradation, glycan biosynthesis and metabolism, tryptophan metabolism, and caprolactam degradation. Several KEGG pathways were also differentially abundant between fermented foods and stool or mouth samples. For example, aminobenzoate degradation, retinol metabolism, naphthalene degradation, ethylbenzene degradation, tyrosine metabolism, and butanoate metabolism pathways were all significantly higher (FDR-corrected *P* value of <0.05) in fermented food samples than in stool samples, while glycosaminoglycan degradation, other glycan degradation, methane metabolism, transcription machinery, sporulation, sphingolipid metabolism, and sporulation pathways were significantly higher in stool samples than in fermented food samples. In mouth samples, *n*-glycan biosynthesis, translation factors and proteins, amino acid-related proteins, and lipopolysaccharide biosynthesis and biosynthesis proteins were significantly (FDR-corrected *P* value of <0.05) higher than in fermented food samples. Conversely, chloroalkane degradation, ethylbenzene degradation, aminobenzoate degradation, tyrosine metabolism, bisphenol degradation, naphthalene degradation, benzoate degradation, xylene degradation, butanoate metabolism, and several other pathways were significantly higher in fermented food samples than in mouth samples.

### Metabolome relationships.

**FIG 3:**
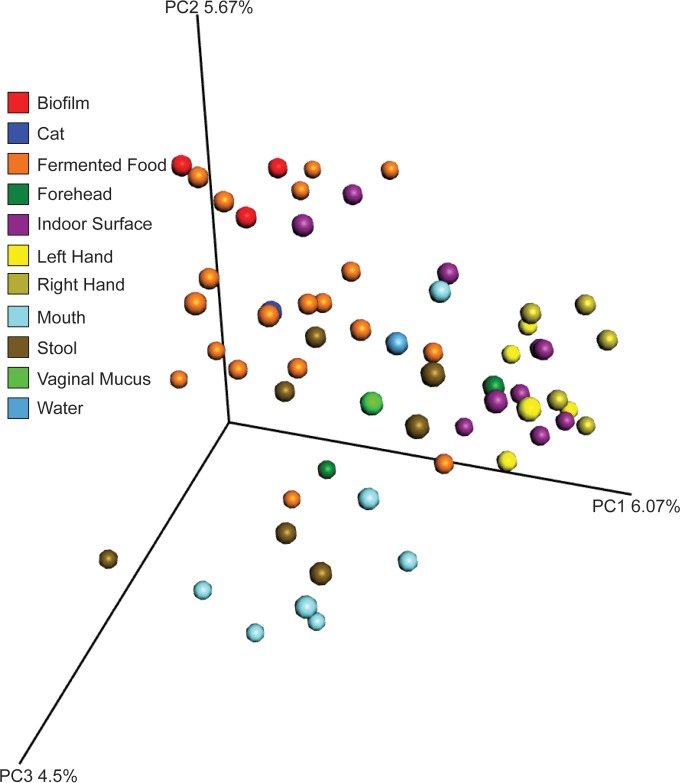
PCoA of the metabolomics data from a presence/absence matrix of unique MS/MS spectra in all samples using the Bray-Curtis distance metric.

PCoA of Bray-Curtis distances for the presence/absence of metabolites by sample showed that skin and mouth samples were distinct from other sample types and that fermented food samples clustered with biofilm samples from their containers (Fig. 3). Stool samples, however, were mixed with other sample types, unlike the tight clustering seen using the 16S rRNA sequencing data (Fig. 3; see also Fig. S1 in the supplemental material). These clustering relationships showed that the chemistry of fermented foods and their associated human and environmental samples was more variable than the microbial profiles among sample types, likely due to the dynamic nature of metabolite production from microbial communities and the direct input of the foods themselves in stool chemistry.

**FIG 4:**
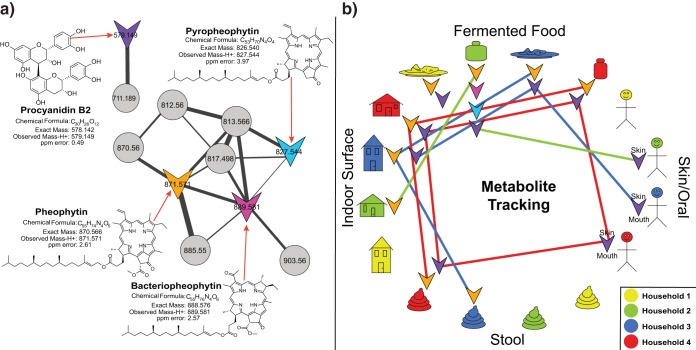
(a) Molecular network clusters of pheophytin and procyanidin and their related metabolites. (b) Metabolite tracking for the presence of those metabolites in the human and environmental samples from the four separate households sampled. Metabolites from network clusters, colored as in panel a, are shown next to the household samples they were detected in, and colored lines are used to visualize tracking of metabolites through the specific households as shown in the key.

Of the 7,425 unique MS/MS spectra detected, 100 were matched to reference libraries using GNPS molecular networking (30, 31). This 1.3% match rate is similar to the 1.8% match rates for all metabolomics data in GNPS (32). Most spectral matches were plant natural products associated with the fermented foods, including flavonoids, lipids, and plant sterols. Other, non-plant-related molecules were observed, including cholesterol and its derivatives on skin and avobenzone, an active ingredient in sunscreen. Gingerol, the spicy flavorant in the ginger root (*Zingiber officinale*), was found in samples of fermented foods and the indoor surfaces of two households. Similarly, the spicy pepper plant (*Piper nigrum*) alkaloid piperine was found in fermented food, stool, indoor surface, and skin samples. The metabolite polanrazine B, isolated from *Leptosphaeria maculans*, a fungal pathogen of canola and rapeseed plants (*Brassica* spp.) (44), was prevalent in two of the four households sampled, including in food and stool samples. Spectral matching also identified the flavonoid procyanidin B2 (*m/z* 579.149), an antioxidant associated with many plants, such as apples, beans, grapes, and tea, and molecular networking detected an altered form with an additional pentose sugar (neutral loss of *m/z* 132.04 [33] [Fig. 4a]). Procyanidin B2 was present in the biofilm, fermented food, indoor surface, human skin, and stool samples. This metabolite was present in all sample types from a single subject, including the foods the person ate, surfaces in the household, the person’s body, and stool (Fig. 4b). Although fermented foods from all four households contained procyanidin B2, only two of them had this molecule in their stool, indicating differential metabolism in different individuals. The modified form of procyanidin (*m/z* 711.189) was found in the same sample types except stool, suggesting that consumption of this metabolite from a fermented food resulted in removal of the sugar or the absorption of the molecule as it passed the digestive tract. Pheophytin A, chlorophyll *a* without its metal ion, was only detected in samples of fermented foods of vegetable origin (except beer), their containers, and stool, indicating that this molecule remained intact through digestion (Fig. 4a and b). Related metabolites, including bacteriopheophytin and pyropheophytin, were detected only in kimchi (Fig. 4a). In sum, analysis of metabolites from human samples revealed molecules from fermented foods modified by human or microbial enzymes, molecules produced by organisms pathogenic for components of the fermented food, molecules from fermented food that passed completely through the volunteers’ digestive tracts without alteration, and differential metabolism of fermented food metabolites in different people.

### Microbiome and metabolome integration.

**FIG 5:**
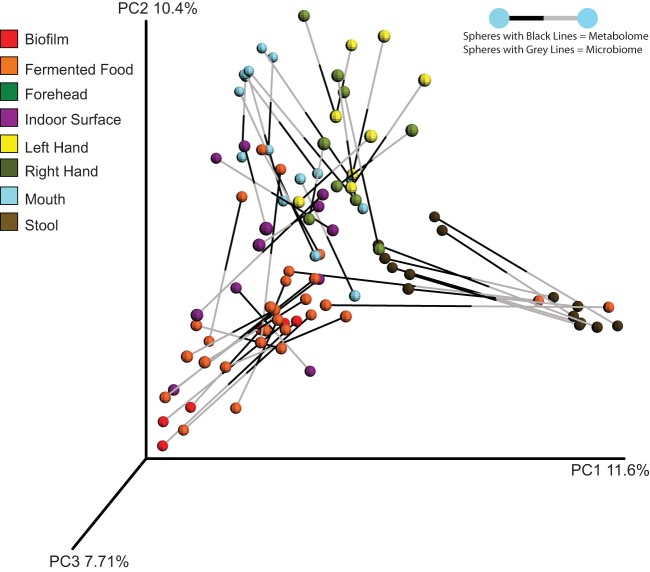
Procrustes analysis of microbiome and metabolome data. Spheres represent individual samples, and they are shown to be either metabolome or microbiome samples by being connected to a grey line or black line, respectively. Connections between the spheres represent microbiomes and metabolomes from the same sample and the distance between them.

Using Procrustes analysis (34) to get an integrated look at metabolome and microbiome relationships, we mapped the principal coordinate analysis matrices of the 16S rRNA data to the metabolomics data. The overall patterns matched, except that two samples (kombucha and pickles) clustered with fecal microbiome samples in the microbiome space but with other fermented foods in the metabolomics space (Fig. 5). These results underscore that microbial communities and their activities are environment specific and that the metabolite output of the sample type is consistent with the microbial community that produced it.

### Conclusions.

Rather than multi-omics analysis being an arduous and highly technical procedure, this study demonstrates that it can be performed on a rapid time scale with a small team of people (six authors of the manuscript contributed to data analysis). A major advantage to this pipeline is the ability to compare data to large data repositories, such as the AGP and GNPS, for sample relationships and metabolite identification. This more easily facilitates the identification of microbiome dysbiosis or metabolome changes that indicate disease. Context is required in any clinical or industrial application of multi-omics data, to better determine how the current structure of a microbial community compares to previous states or sample types, enabling diagnosis of an active dysbiosis. The present study focused on fermented foods and their effects on the people who prepared and consumed them. These foods are of enormous medical importance given that yogurt, a fermented food, is the single food most correlated epidemiologically with weight loss in the U.S. population (35), and they are of economic importance due to the billions of dollars per year that fermented foods contribute to the economy. Although this sample cohort did not require rapid data analysis, such as that required in a medical emergency or the potential loss of a large industrial fermentation, this study shows that consent could be obtained, samples collected, and data generated on microbiome-related samples collected from people located up to 100 miles away from the laboratory in a time frame matching that of classic microbiological culturing of common pathogens (approximately 2 days). The ability to do rapid-response multi-omics analysis and systems biology will have far reaching implications, from monitoring industrial fermentation processes, to guiding oil and gas drilling and fracking decisions, to providing rapid molecular analysis for patient care in infectious diseases and guiding the use of microbiome-based therapies, such as fecal microbiota transplant (FMT) (36) and probiotics. The combination of standardized protocols for subject recruitment and consent, sample collection, metadata capture, DNA sequencing, mass spectrometry, molecular networking, and data analysis and visualization now puts this technology in the hands of a broad spectrum of users. Broader and more rapid use of multi-omics methods will begin a sea change towards their implementation in clinical medicine.

## MATERIALS AND METHODS

### Participant recruitment and sample collection.

For the first application of the pipeline, we chose a situation that, while time sensitive, was not necessary for clinical decisions. All participants are members of a local fermenter’s club and ferment at home or operate a fermented food business; they learned about the study through the fermenter’s club. Participants willing to sample their own bodies, their fermented foods, and the surfaces that their foods are prepared on or in (i.e., kitchen counters, cutting boards, and fermenters) consented to be a part of the American Gut Project (AGP), the largest crowd-sourced, crowd-funded citizen science project in existence today. A total of seven people (two families and two individuals, designated households 1 to 4) received barcoded, dual-headed sterile cotton sampling swabs (BD Swube; Becton, Dickinson and Company, Franklin Lakes, NJ) and were instructed to sample their skin (right and left hands), mouths, stool, their fermented foods, and the surfaces touched by those foods. Some participants chose to sample alternative body sites (i.e., vagina and forehead), and one participant sampled the mouth of a pet cat. The food samples collected included beer, port wine, pickled cucumbers, pickled jalapenos, cottage cheese, curtido, kefir, kimchi, sauerkraut, miso, beet kvass, and fermented soda (see Table S1 in the supplemental material). The surface samples collected included cutting boards, countertops, refrigerator surfaces, skillets, kegerator parts, and fermentor parts. Samples were collected by subjects on 25, 26, and 27 January 2016, with the first sample in the data set collected at 8:05 a.m. on 25 January and the last sample in the data set collected at 12:05 p.m. on 27 January, for a total of 61 samples. Samples from six participants were delivered by hand to the laboratory, while one participant mailed their samples to the laboratory via overnight priority mail (FedEx). All samples were received in the laboratory by 1:07 p.m. on 27 January 2016 (Fig. 1). Upon arrival, one swab head from each dual-headed swab was immediately placed into a MoBio PowerSoil DNA extraction kit bead plate (MoBio, Inc., Carlsbad, CA) for bacterial DNA extraction. The second swab head was stored overnight at −20°C before preparation for metabolomics analysis using mass spectrometry.

### Bacterial DNA extraction and generation of 16S rRNA V4 amplicons.

Bacterial genomic DNA extraction, 16S rRNA gene variable region 4 (V4) amplicon generation, and amplicon preparation for sequencing were performed according to protocols benchmarked for the Earth Microbiome Project (EMP) that can be found on the EMP website (http://www.earthmicrobiome.org/emp-standard-protocols/). Briefly, bacterial genomic DNA was extracted from samples using the PowerMag DNA isolation kit optimized for KingFisher (Mo Bio Laboratories, Carlsbad, CA), and then the V4 region of the 16S rRNA gene was amplified in triplicate from each sample and combined as follows. The PCR mixtures contained 13 µl Mo Bio PCR water, 10 µl 5 Prime HotMasterMix, 0.5 µl each of the barcoded forward and reverse primers (515f and 806rB; 10 µM final concentration), and 1.0 µl genomic DNA. The reaction mixtures were held at 94°C for 3 min (denaturation), with amplification proceeding for 35 cycles at 94°C for 45 s, 50°C for 60 s, and 72°C for 90 s, followed by a final extension for 10 min at 72°C. After amplification, the DNA concentration was quantified using PicoGreen double-stranded DNA (dsDNA) reagent in 10 mM Tris buffer (pH 8.0). A composite sample for sequencing was created by combining equimolar ratios of amplicons from the individual samples, followed by ethanol precipitation to remove any remaining contaminants and PCR artifacts.

### 16S rRNA marker gene sequencing.

Pooled amplicons were sequenced at the Institute for Genomic Medicine at the University of California, San Diego, using the Illumina MiSeq platform. The library concentration was measured using the HiSens Qubit dsDNA HS assay kit (Thermo Fisher Scientific). A total of 6 pM of 16S library combined with 0.9 pM (15%) PhiX sequencing control version 3 was sequenced with 150-bp paired-end (PE) reads on an Illumina MiSeq sequencing system using a MiSeq reagent kit version 2 (300 cycle). Fastq files for reads 1 and 2 and the index read were generated using the BCL-to-FASTQ file converter bcl2fastq version 2.17.1.14 (Illumina, Inc.).

### 16S rRNA marker gene data analysis.

Sequencing data were prepared and analyzed using the online tool Qiita (https://qiita.microbio.me) and the QIIME pipeline (37) version 1.9. Illumina read 1 was quality filtered and demultiplexed according to the QIIME default parameters, as follows: no ambiguous bases allowed, only one bar code mismatch allowed, and a minimum required Phred quality score of 3. Quality filtering resulted in 6,830,655 high-quality reads, with the average number of sequences per sample being 84,329. Quality-filtered sequences were clustered using the closed-reference OTU picking workflow against the August 2013 release of the Greengenes database (DeSantis et al. [38]), with a sequence identity of 97% and sortmeRNA (39) as the underlying clustering algorithm. After OTU picking, 5 samples (forehead, water, vaginal, fermented grape soda, and fermenter inner wall samples) were removed from the data set because they had sequence counts lower than the rarefaction cutoff (2,053 sequences per sample); thus, a total of 54 microbiome samples were included in downstream analyses.

The AGP team has identified a group of bacterial bloom sequences that increase during sample transit back to the laboratory, and in order to avoid a study bias, those sequences were filtered out of the data (code available at https://github.com/biocore/American-Gut/blob/master/ipynb/primary-processing/02-filter_sequences_for_blooms.md). To facilitate direct comparisons and reduce study bias between data obtained from the fermentation cohort and the AGP cohort, fermentation cohort stool sample data were also filtered for blooms.

Five of the seven fecal samples from the fermentation cohort passed quality and sequencing depth filtering. The bacterial diversity levels observed in these five samples were compared to those in a subset of 122 randomly selected fecal samples from other AGP participants of a similar age group for whom data on the frequency of fermented food intake were available. Alpha diversity (measured as Faith’s phylogenetic diversity [26]) was calculated for each sample from a rarefied OTU table of 2,053 sequences per sample. Barplots were generated in R (https://www.r-project.org/) to visualize the distribution of diversity values across the various groups, and a linear regression model was fitted to the AGP portion of the data.

We used SourceTracker (29), a tool that uses a Bayesian model jointly with Gibbs sampling to quantify the amount of taxa that a set of source environments contributes to a sink environment, to determine the proportions of human and surface microbes that were sourced from fermented food microbiomes. Fermented food samples were designated “sources,” while human and surface samples were designated “sinks.”

Statistical analyses were applied to determine the significance of groups by sample type on the PCoA plot (ANOSIM, 999 Monte Carlo permutations) and to identify OTUs with significantly different relative abundances (Kruskal-Wallis, 999 Monte Carlo permutations) across sample groups. Nonparametric tests were used to appropriately deal with microbiome data, which were not normally distributed. The significance cutoff for *P* values (ANOSIM) and FDR-corrected *P* values (Kruskal-Wallis) was set at 0.05.

PICRUSt metagenome predictions were performed using the Galaxy implementation of PICRUSt 1.0.0 (40). The resulting BIOM table was then categorized by KEGG pathways (i.e., KEGG Orthology groups [KOs] were placed into functional categories). All eukaryote-specific pathways were removed from the table, and the table was rarefied to 572,338. The Bray-Curtis distance metric was then applied and visualized using EMPeror (34). A Kruskall-Wallis test with 999 Monte Carlo permutations was applied to determine significant differences in KEGG pathway abundances between groups of samples.

### Metabolomics data analysis.

The metabolomics data for this project are available under MassIVE data set ID MSV000079485 at http://gnps.ucsd.edu. To generate metabolomes, the swabs were added to a solution of 70% methanol in water and allowed to extract for 2 h at room temperature. The methanol extract was then dried down in a centrifugal evaporator and redissolved in 100% methanol. Samples were transferred into 2-ml vials with inserts and diluted 1:2. MS analysis was performed on a QExactive (Thermo Scientific) mass spectrometer with a heated electrospray ionization (HESI-II) probe source, controlled by Xcalibur 3.0 software. MS spectra were acquired in positive ion mode over a mass range of 100 to 1,500 m*/z*. An external calibration with Pierce LTQ Velos electrospray ionization (ESI) positive ion calibration solution (Thermo Scientific) was performed prior to data acquisition, with an error rate of less than 1 ppm. The following probe settings were used for flow aspiration and ionization: spray voltage of 3,500 V, sheath gas (N_2_) pressure of 53 lb/in^2^, auxiliary gas (N_2_) pressure of 14 lb/in^2^, ion source temperature of 270°C, S-lens radio frequency (RF) level of 50 Hz, and auxiliary gas heater temperature at 440°C. Data acquisition parameters were as follows. Minutes 0 to 0.5 were sent to waste. Minutes 0.5 to 12 were recorded with data-dependent MS/MS acquisition mode. Full scan at MS^1^ level was performed with resolution of 35,000 in profile mode. The 10 most intense ions with 1 *m*/*z* isolation window per MS^1^ scan were selected and subjected to normalized collision-induced dissociation with 30 eV. MS^2^ scans were performed at 17,500 resolution with maximum injection time of 60 ms in profile mode. The MS/MS active exclusion parameter was set to 5.0 s. The injected samples were chromatographically separated using a Vanquish ultrahigh-performance liquid chromatography (UHPLC) instrument (Thermo Scientific) controlled by Thermo SII for Xcalibur software (Thermo Scientific), with a 30- by 2.1-mm, 2.6 µM, C_18_, 100-Å Kinetex chromatography column (Phenomenex) with 40°C column temperature, 0.5 ml/min flow rate, mobile phase A consisting of 99.9% water (LC-MS grade; J.T. Baker)–0.1% formic acid (Fisher Scientific, Optima LC/MS), and mobile phase B consisting of 99.9% acetonitrile (LC-MS grade; J.T. Baker)–0.1% formic acid (Fisher Scientific, Optima LC/MS), using the following gradient: 0 to 1 min, 5% B; 1 to 8 min, 100% B; 8 to 10.9 min, 100% B; 10.9 to 11 min, 5% A; and 11 to 12 min, 5% B. Raw data files were converted to the .mzXML format using ProteoWizard (http://proteowizard.sourceforge.net/) and uploaded to the GNPS-MassIVE mass spectrometry database. The list of annotations from the search can be found at http://gnps.ucsd.edu/ProteoSAFe/result.jsp?task=efc4f1031f73471cbdfddcde0cc181a6&view=view_all_annotations_DB.

Molecular networking was performed to identify spectra shared between different sample types and to identify known molecules in the data set. All annotations are at level 2 according to the proposed minimum standards in metabolomics (41). The molecular networking parameters were as follows: a minimum matched-peak threshold of 4, a cosine similarity score cutoff of 0.65, a minimum cluster size of 2, and a parent and ion tolerance of 0.5 Da. GNPS library search parameters were the same except that a cosine threshold of 0.7 was used. A feature table of metabolite presence and absence in each sample was generated from GNPS spectral alignments and downloaded. Similarity of metabolomes was determined using the Bray-Curtis distance metric, projected with principal coordinate analysis and visualized with EMPeror (Fig. 1) through the in-house tool ClusterApp. Molecular networks were visualized and mined using the Cytoscape software (42).

### 16S-metabolomics multivariate comparisons.

Using the OTU table and the metabolite table, we generated a distance matrix for each, using unweighted UniFrac for 16S and Bray-Curtis for the metabolomics. We performed principal coordinate analysis on the two matrices separately and used Procrustes analysis as implemented in QIIME 1.9.1 to rotate, translate, and scale the matrices. The resulting transformed matrices were plotted using EMPeror (34).

### Microarray data accession numbers.

Mapping files and preprocessed data for human samples are available at https://qiita.ucsd.edu under Qiita study identification number (ID) 10317 (AGP), and sequences are publicly available in EMBL-EBI (accession number ERP012803) under accession numbers ERS1048817, ERS1048818, ERS1048819, ERS1048820, ERS1048821, ERS1048822, ERS1048823, ERS1048824, ERS1048825, ERS1048826, ERS1048827, ERS1048828, ERS1048829, ERS1048832, ERS1048833, ERS1048834, ERS1048835, ERS1048836, ERS1048837, ERS1048838, ERS1048839, ERS1048840, ERS1048841, ERS1048842, ERS1048843, ERS1048844, and ERS1048845. Mapping files and preprocessed data for food, environment, and cat samples are available at https://qiita.ucsd.edu under Qiita study ID 10395, and sequences are publicly available in EMBL-EBI (accession number ERP015077). The 16S amplicon analyses outlined in this paper were conducted using the Knight laboratory’s supercomputer Barnacle, using 26 CPU hours.

## References

[1] Read TD, Salzberg SL, Pop M, Shumway M, Umayam L, Jiang L, Holtzapple E, Busch JD, Smith KL, Schupp JM, Solomon D, Keim P, Fraser CM 2002 Comparative genome sequencing for discovery of novel polymorphisms in Bacillus anthracis. Science 296:2028–2033. doi:10.1126/science.1071837. 12004073

[2] Mellmann A, Harmsen D, Cummings CA, Zentz EB, Leopold SR, Rico A, Prior K, Szczepanowski R, Ji Y, Zhang W, McLaughlin SF, Henkhaus JK, Leopold B, Bielaszewska M, Prager R, Brzoska PM, Moore RL, Guenther S, Rothberg JM, Karch H 2011 Prospective genomic characterization of the German enterohemorrhagic Escherichia coli O104:H4 outbreak by rapid next generation sequencing technology. PLoS One 6:e22751. doi:10.1371/journal.pone.0022751. 21799941 PMC3140518

[3] Naccache SN, Federman S, Veeraraghavan N, Zaharia M, Lee D, Samayoa E, Bouquet J, Greninger AL, Luk K-C, Enge B, Wadford DA, Messenger SL, Genrich GL, Pellegrino K, Grard G, Leroy E, Schneider BS, Fair JN, Martínez MA, Isa P, Crump JA, DeRisi JL, Sittler T, Hackett J, Miller S, Chiu CY 2014 A cloud-compatible bioinformatics pipeline for ultrarapid pathogen identification from next-generation sequencing of clinical samples. Genome Res 24:1180–1192. doi:10.1101/gr.171934.113. 24899342 PMC4079973

[4] Greninger AL, Naccache SN, Federman S, Yu G, Mbala P, Bres V, Stryke D, Bouquet J, Somasekar S, Linnen JM, Dodd R, Mulembakani P, Schneider BS, Muyembe-Tamfum J-J, Stramer SL, Chiu CY 2015 Rapid metagenomic identification of viral pathogens in clinical samples by real-time nanopore sequencing analysis. Genome Med 7:99. doi:10.1186/s13073-015-0220-9. 26416663 PMC4587849

[5] Quick J, Loman NJ, Duraffour S, Simpson JT, Severi E, Cowley L, Bore JA, Koundouno R, Dudas G, Mikhail A, Ouédraogo N, Afrough B, Bah A, Baum JHJ, Becker-Ziaja B, Boettcher JP, Cabeza-Cabrerizo M, Camino-Sánchez Á, Carter LL, Doerrbecker J, Enkirch T, Dorival IG, Hetzelt N, Hinzmann J, Holm T, Kafetzopoulou LE, Koropogui M, Kosgey A, Kuisma E, Logue CH, Mazzarelli A, Meisel S, Mertens M, Michel J, Ngabo D, Nitzsche K, Pallasch E, Patrono LV, Portmann J, Repits JG, Rickett NY, Sachse A, Singethan K, Vitoriano I, Yemanaberhan RL, Zekeng EG, Racine T, Bello A, Sall AA, Faye O, et al. 2016 Real-time, portable genome sequencing for *Ebola* surveillance. Nature 530:228–232. doi:10.1038/nature16996. 26840485 PMC4817224

[6] Balog J, Sasi-Szabó L, Kinross J, Lewis MR, Muirhead LJ, Veselkov K, Mirnezami R, Dezső B, Damjanovich L, Darzi A, Nicholson JK, Takáts Z 2013 Intraoperative tissue identification using rapid evaporative ionization mass spectrometry. Sci Transl Med 5:194ra93. doi:10.1126/scitranslmed.3005623. 23863833

[7] Hsu C-C, Elnaggar MS, Peng Y, Fang J, Sanchez LM, Mascuch SJ, Møller KA, Alazzeh EK, Pikula J, Quinn RA, Zeng Y, Wolfe BE, Dutton RJ, Gerwick L, Zhang L, Liu X, Månsson M, Dorrestein PC 2013 Real-time metabolomics on living microorganisms using ambient electrospray ionization flow-probe. Anal Chem 85:7014–7018. doi:10.1021/ac401613x. 23819546 PMC3890442

[8] Fritz JV, Desai MS, Shah P, Schneider JG, Wilmes P 2013 From meta-omics to causality: experimental models for human microbiome research. Microbiome 1:14. doi:10.1186/2049-2618-1-14. 24450613 PMC3971605

[9] Franzosa EA, Hsu T, Sirota-Madi A, Shafquat A, Abu-Ali G, Morgan XC, Huttenhower C 2015 Sequencing and beyond: integrating molecular “omics” for microbial community profiling. Nat Rev Microbiol 13:360–372. doi:10.1038/nrmicro3451. 25915636 PMC4800835

[10] Wang Z, Klipfell E, Bennett BJ, Koeth R, Levison BS, Dugar B, Feldstein AE, Britt EB, Fu X, Chung Y-M, Wu Y, Schauer P, Smith JD, Allayee H, Tang WH, DiDonato JA, Lusis AJ, Hazen SL 2011 Gut flora metabolism of phosphatidylcholine promotes cardiovascular disease. Nature 472:57–63. doi:10.1038/nature09922. 21475195 PMC3086762

[11] Koeth RA, Wang Z, Levison BS, Buffa JA, Org E, Sheehy BT, Britt EB, Fu X, Wu Y, Li L, Smith JD, DiDonato JA, Chen J, Li H, Wu GD, Lewis JD, Warrier M, Brown JM, Krauss RM, Tang WH, Bushman FD, Lusis AJ, Hazen SL 2013 Intestinal microbiota metabolism of L-carnitine, a nutrient in red meat, promotes atherosclerosis. Nat Med 19:576–585. doi:10.1038/nm.3145. 23563705 PMC3650111

[12] Haiser HJ, Gootenberg DB, Chatman K, Sirasani G, Balskus EP, Turnbaugh PJ 2013 Predicting and manipulating cardiac drug inactivation by the human gut bacterium Eggerthella lenta. Science 341:295–298. doi:10.1126/science.1235872. 23869020 PMC3736355

[13] Maurice CF, Haiser HJ, Turnbaugh PJ 2013 Xenobiotics shape the physiology and gene expression of the active human gut microbiome. Cell 152:39–50. doi:10.1016/j.cell.2012.10.052. 23332745 PMC3552296

[14] Li M, Wang B, Zhang M, Rantalainen M, Wang S, Zhou H, Zhang Y, Shen J, Pang X, Wei H, Chen Y, Lu H, Zuo J, Su M, Qiu Y, Jia W, Xiao C, Smith LM, Yang S, Holmes E, Tang H, Zhao G, Nicholson JK, Li L, Zhao L 2008 Symbiotic gut microbes modulate human metabolic phenotypes. Proc Natl Acad Sci U S A 105:2117–2122. doi:10.1073/pnas.0712038105. 18252821 PMC2538887

[15] Smith MI, Yatsunenko T, Manary MJ, Trehan I, Mkakosya R, Cheng J, Kau AL, Rich SS, Concannon P, Mychaleckyj JC, Liu J, Houpt E, Li JV, Holmes E, Nicholson J, Knights D, Ursell LK, Knight R, Gordon JI 2013 Gut microbiomes of Malawian twin pairs discordant for kwashiorkor. Science 339:548–554. doi:10.1126/science.1229000. 23363771 PMC3667500

[16] Ridaura VK, Faith JJ, Rey FE, Cheng J, Duncan AE, Kau AL, Griffin NW, Lombard V, Henrissat B, Bain JR, Muehlbauer MJ, Ilkayeva O, Semenkovich CF, Funai K, Hayashi DK, Lyle BJ, Martini MC, Ursell LK, Clemente JC, Van Treuren W 2013 Gut microbiota from twins discordant for obesity modulate metabolism in mice Science 341:1241214. doi:10.1126/science.1241214. 24009397 PMC3829625

[17] McGovern PE 2003 Ancient wine: the search for the origin of viticulture. Princeton University Press, Princeton, NJ.

[18] Wolfe BE, Dutton RJ 2015 Fermented foods as experimentally tractable microbial ecosystems. Cell 161:49–55. doi:10.1016/j.cell.2015.02.034. 25815984

[19] Van Hylckama Vlieg JE, Veiga P, Zhang C, Derrien M, Zhao L 2011 Impact of microbial transformation of food on health—from fermented foods to fermentation in the gastro-intestinal tract. Curr Opin Biotechnol 22:211–219. doi:10.1016/j.copbio.2010.12.004. 21247750

[20] David LA, Maurice CF, Carmody RN, Gootenberg DB, Button JE, Wolfe BE, Ling AV, Devlin AS, Varma Y, Fischbach MA, Biddinger SB, Dutton RJ, Turnbaugh PJ 2014 Diet rapidly and reproducibly alters the human gut microbiome. Nature 505:559–563. doi:10.1038/nature12820. 24336217 PMC3957428

[21] Turnbaugh PJ, Ridaura VK, Faith JJ, Rey FE, Knight R, Gordon JI 2009 The effect of diet on the human gut microbiome: a metagenomic analysis in humanized gnotobiotic mice. Sci Transl Med 1:6ra14. doi:10.1126/scitranslmed.3000322. PMC2894525 20368178

[22] Caporaso JG, Lauber CL, Walters WA, Berg-Lyons D, Huntley J, Fierer N, Owens SM, Betley J, Fraser L, Bauer M, Gormley N, Gilbert JA, Smith G, Knight R 2012 Ultra-high-throughput microbial community analysis on the Illumina HiSeq and MiSeq platforms. ISME J 6:1621–1624. doi:10.1038/ismej.2012.8. 22402401 PMC3400413

[23] Knight R, Jansson J, Field D, Fierer N, Desai N, Fuhrman JA, Hugenholtz P, van der Lelie D, Meyer F, Stevens R, Bailey MJ, Gordon JI, Kowalchuk GA, Gilbert JA 2012 Unlocking the potential of metagenomics through replicated experimental design. Nat Biotechnol 30:513–520. doi:10.1038/nbt.2235. 22678395 PMC4902277

[24] Bouslimani A, Porto C, Rath CM, Wang M, Guo Y, Gonzalez A, Berg-Lyon D, Ackermann G, Moeller Christensen GJ, Nakatsuji T, Zhang L, Borkowski AW, Meehan MJ, Dorrestein K, Gallo RL, Bandeira N, Knight R, Alexandrov T, Dorrestein PC 2015 Molecular cartography of the human skin surface in 3D. Proc Natl Acad Sci U S A 112:E2120–E2129. doi:10.1073/pnas.1424409112. 25825778 PMC4418856

[25] Vinaixa M, Schymanski EL, Neumann S, Navarro M, Salek RM, Yanes O 2016 Mass spectral databases for LC/MS and GC/MS-based metabolomics: state of the field and future prospects. Trends Analyt Chem 78:23–35. doi:10.1016/j.trac.2015.09.005.

[26] Faith DP 1992 Conservation evaluation and phylogenetic diversity. Biol Conserv 61:1–10. doi:10.1016/0006-3207(92)91201-3.

[27] Lozupone C, Knight R 2005 UniFrac: a new phylogenetic method for comparing microbial communities. Appl Environ Microbiol 71:8228–8235. doi:10.1128/AEM.71.12.8228-8235.2005. 16332807 PMC1317376

[28] Human Microbiome Project Consortium. 2012 Structure, function and diversity of the healthy human microbiome. Nature 486:207–214. doi:10.1038/nature11234. 22699609 PMC3564958

[29] Knights D, Kuczynski J, Charlson ES, Zaneveld J, Mozer MC, Collman RG, Bushman FD, Knight R, Kelley ST 2011 Bayesian community-wide culture-independent microbial source tracking. Nat Methods 8:761–763. doi:10.1038/nmeth.1650. 21765408 PMC3791591

[30] Watrous J, Roach P, Alexandrov T, Heath BS, Yang JY, Kersten RD, van der Voort M, Pogliano K, Gross H, Raaijmakers JM, Moore BS, Laskin J, Bandeira N, Dorrestein PC 2012 Mass spectral molecular networking of living microbial colonies. Proc Natl Acad Sci U S A 109:E1743–E1752. doi:10.1073/pnas.1203689109. 22586093 PMC3387089

[31] Yang JY, Sanchez LM, Rath CM, Liu X, Boudreau PD, Bruns N, Glukhov E, Wodtke A, de Felicio R, Fenner A, Wong WR, Linington RG, Zhang L, Debonsi HM, Gerwick WH, Dorrestein PC 2013 Molecular networking as a dereplication strategy. J Nat Prod 76:1686–1699. doi:10.1021/np400413s. 24025162 PMC3936340

[32] Da Silva RR, Dorrestein PC, Quinn RA 2015 Illuminating the dark matter in metabolomics. Proc Natl Acad Sci U S A 112:12549–12550. doi:10.1073/pnas.1516878112. 26430243 PMC4611607

[33] Prasain JK, Jones K, Kirk M, Wilson L, Smith-Johnson M, Weaver C, Barnes S 2003 Profiling and quantification of isoflavonoids in kudzu dietary supplements by high-performance liquid chromatography and electrospray ionization tandem mass spectrometry. J Agric Food Chem 51:4213–4218. doi:10.1021/jf030174a. 12848487

[34] Vázquez-Baeza Y, Pirrung M, Gonzalez A, Knight R 2013 EMPeror: a tool for visualizing high-throughput microbial community data. Gigascience 2:16. doi:10.1186/2047-217X-2-16. 24280061 PMC4076506

[35] Mozaffarian D, Hao T, Rimm EB, Willett WC, Hu FB 2011 Changes in diet and lifestyle and long-term weight gain in women and men. N Engl J Med 364:2392–2404. doi:10.1056/NEJMoa1014296. 21696306 PMC3151731

[36] Rossen NG, MacDonald JK, de Vries EM, D’Haens GR, de Vos WM, Zoetendal EG, Ponsioen CY 2015 Fecal microbiota transplantation as novel therapy in gastroenterology: a systematic review. World J Gastroenterol 21:5359–5371. doi:10.3748/wjg.v21.i17.5359. 25954111 PMC4419078

[37] Caporaso JG, Kuczynski J, Stombaugh J, Bittinger K, Bushman FD, Costello EK, Fierer N, Peña AG, Goodrich JK, Gordon JI, Huttley GA, Kelley ST, Knights D, Koenig JE, Ley RE, Lozupone CA, McDonald D, Muegge BD, Pirrung M, Reeder J, Sevinsky JR, Turnbaugh PJ, Walters WA, Widmann J, Yatsunenko T, Zaneveld J, Knight R 2010 QIIME allows analysis of high-throughput community sequencing data. Nat Methods 7:335–336. doi:10.1038/nmeth.f.303. 20383131 PMC3156573

[38] DeSantis TZ, Hugenholtz P, Larsen N, Rojas M, Brodie EL, Keller K, Huber T, Dalevi D, Hu P, Andersen GL 2006 Greengenes, a chimera-checked 16S rRNA gene database and workbench compatible with ARB. Appl Environ Microbiol 72:5069–5072. doi:10.1128/AEM.03006-05. 16820507 PMC1489311

[39] Kopylova E, Noé L, Touzet H 2012 SortMeRNA: fast and accurate filtering of ribosomal RNAs in metatranscriptomic data. Bioinformatics 28:3211–3217. doi:10.1093/bioinformatics/bts611. 23071270

[40] Langille MG, Zaneveld J, Caporaso JG, McDonald D, Knights D, Reyes JA, Clemente JC, Burkepile DE, Vega Thurber RL, Knight R, Beiko RG, Huttenhower C 2013 Predictive functional profiling of microbial communities using 16S rRNA marker gene sequences. Nat Biotechnol 31:814–821. doi:10.1038/nbt.2676. 23975157 PMC3819121

[41] Sumner LW, Amberg A, Barrett D, Beale MH, Beger R, Daykin CA, Fan TW-M, Fiehn O, Goodacre R, Griffin JL, Hankemeier T, Hardy N, Harnly J, Higashi R, Kopka J, Lane AN, Lindon JC, Marriott P, Nicholls AW, Reily MD, Thaden JJ, Viant MR 2007 Proposed minimum reporting standards for chemical analysis: Chemical Analysis Working Group (CAWG) metabolomics Standards Initiative (MSI). Metabolomics 3:211–221. doi:10.1007/s11306-007-0082-2. 24039616 PMC3772505

[42] Shannon P, Markiel A, Ozier O, Baliga NS, Wang JT, Ramage D, Amin N, Schwikowski B, Ideker T 2003 Cytoscape: a software environment for integrated models of biomolecular interaction networks. Genome Res 13:2498–2504. doi:10.1101/gr.1239303. 14597658 PMC403769

[43] Towns J, Cockerill T, Dahan M, Foster I, Gaither K, Grimshaw A, Hazlewood V, Lathrop S, Lifka D, Peterson GD, Roskies R, Scott JR, Wilkens-Diehr N 2014 XSEDE: accelerating scientific discovery. Comput Sci Eng 16:62–74. doi:10.1109/MCSE.2014.80.

[44] Sprague SJ, Watt M, Kirkegaard JA, Howlett BJ 2007 Pathways of infection of Brassica napus roots by Leptosphaeria maculans. New Phytol 176:211–222. doi:10.1111/j.1469-8137.2007.02156.x. 17696980

