# Supplementary material for: From Sample to Multi-Omics Conclusions in under 48 Hours
Source: mSystems. 2016 Apr 26;1(2):e00038-16. doi: 10.1128/mSystems.00038-16 (PMC5069746; doi:10.1128/mSystems.00038-16)
Supplement: Table S1 [file sys002162017st2.docx]

Table S1. Specific fermented foods produced and consumed by household.

| Household | Fermented Foods |
| --- | --- |
| 1 | Kombucha, Beet, Kvass, Grape Soda |
| 2 | Kimchi, Curtido, Water Kefir, Sauerkraut, Miso, Dairy Kefir |
| 3 | Beer, Port Wine, Pickled Jalapenos, Beer, Cottage Cheese, Pickles |
| 4 | Kombucha, Kombucha, SCOBY |
