# Supplementary material for: From Sample to Multi-Omics Conclusions in under 48 Hours
Source: mSystems. 2016 Apr 26;1(2):e00038-16. doi: 10.1128/mSystems.00038-16 (PMC5069746; doi:10.1128/mSystems.00038-16)

Figure S1. PCoA plot of 16S marker gene sequencing from food, human and environmental samples collected in this study.

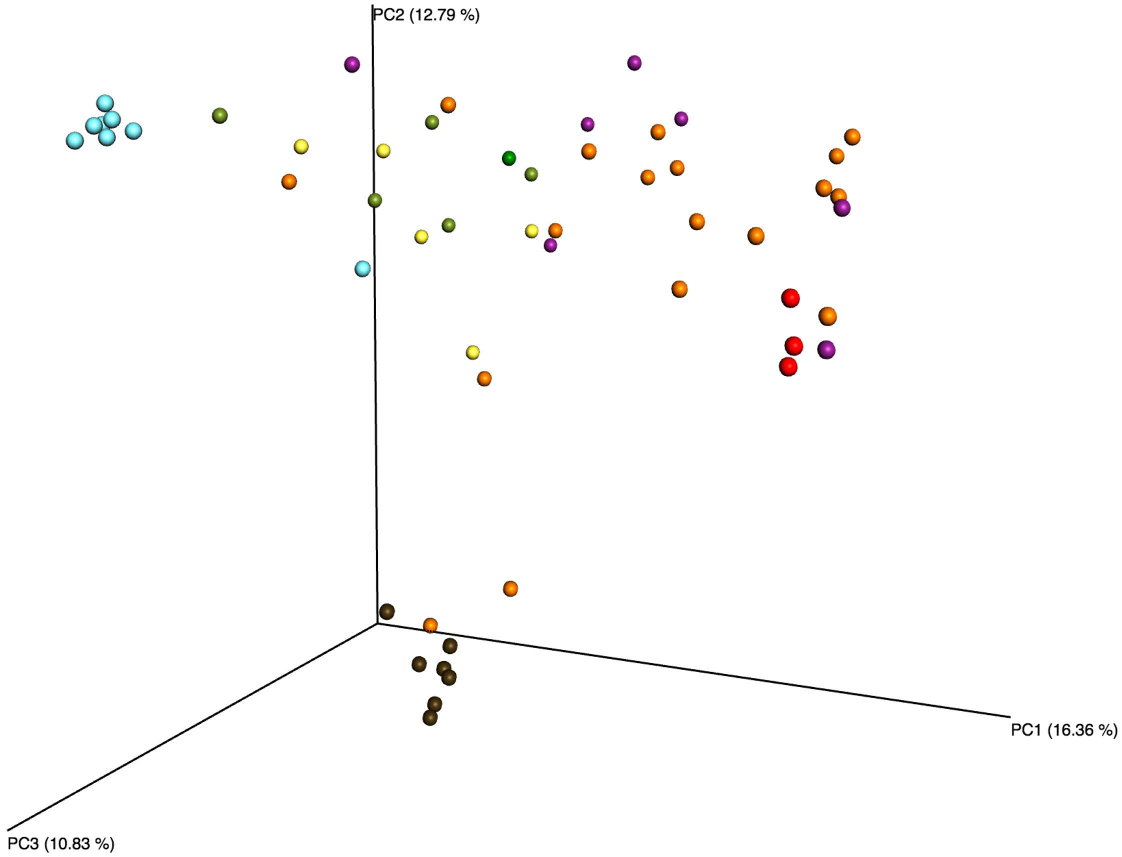

Supplement: Figure S1 [file sys002162017sf1.pdf]
